# IFN signaling at the nexus of the radiotherapy response in malignant peripheral nerve sheath tumors

**DOI:** 10.1172/JCI202266

**Published:** 2026-03-02

**Authors:** Sean P. Pitroda, Ralph R. Weichselbaum

**Affiliations:** Department of Radiation and Cellular Oncology, The University of Chicago, Chicago, Illinois, USA.

## Abstract

Malignant peripheral nerve sheath tumors (MPNSTs) are aggressive sarcomas that constitute a major cause of mortality in individuals with neurofibromatosis type 1 (NF-1) and exhibit highly variable responses to radiotherapy. In this issue of the *JCI*, Zhu and colleagues integrated functional genomics, single-cell transcriptomics, and analysis of human tumors to show that type I IFN signaling shapes both tumor-intrinsic radiation sensitivity of MPNSTs and local recruitment and activation of T cells. Their findings establish IFN signaling as a central coordinator of the radiotherapy response in MPNSTs and suggest that incorporating targeted immunomodulation strategies may improve radiotherapy outcomes. The work also has direct implications for the role of the immune system and IFN signaling radiation–based treatment of soft tissue sarcomas beyond those involved in NF-1.

## Radiotherapy in malignant peripheral nerve sheath tumors

Malignant peripheral nerve sheath tumors (MPNSTs) are aggressive soft tissue sarcomas that arise from peripheral nerve sheaths and carry particularly adverse outcomes for patients with neurofibromatosis type 1 (NF-1). These tumors represent a leading cause of early mortality for patients with NF-1 (1) and remain among the most challenging tumors to treat effectively. In patients with NF-1, the therapeutic window for radiotherapy is complex, and there is substantial heterogeneity in how individual MPNSTs respond to radiation. Certain tumors demonstrate marked regression, whereas others progress despite optimal dosing and technique. Until now, we have lacked a biological framework to explain this variability in response or predict which patients are most likely to benefit from treatment.

Zhu and colleagues have addressed this gap by identifying type I IFN signaling as a key determinant of radiation response in MPNSTs (2). Rather than serving simply as a generic inflammation marker or downstream consequence of cellular stress, IFN signaling appears to function as a coordinating mechanism that links tumor stress responses directly to immune engagement.

## A radiation-induced IFN state that distinguishes malignancy

In the foundational experiments of this study, the authors demonstrated that MPNST cells, but not benign neurofibroma cells, mounted a robust type I IFN transcriptional program following irradiation. This distinction is worth emphasizing: the difference observed was not simply that malignant cells had higher baseline IFN signaling than did their benign counterparts. Rather, the IFN response emerged specifically in response to radiation-induced damage. This suggests that malignant transformation in this lineage involves acquisition of the machinery to detect and respond to genotoxic stress through IFN pathways.

To uncover the IFN response, Zhu et al. conducted a genome-wide CRISPR interference screen on two human MPNST cell lines to identify genes functionally required for radiation sensitivity (2). Genes encoding STING (*TMEM173*), IRF3, IFNAR1, and STAT1 all scored as critical determinants of radiosensitivity. These proteins represent the core signaling axis of the type I IFN response. Loss of function in any of these genes reduced radiation sensitivity, establishing that IFN signaling is part of the operational circuitry that determines how MPNST cells respond to radiation, not merely a correlative biomarker. Interestingly, these observations appear to be at odds with some previously published findings, which we will discuss below.

## The radiation response depends on immune cell engagement

**Figure 1:**
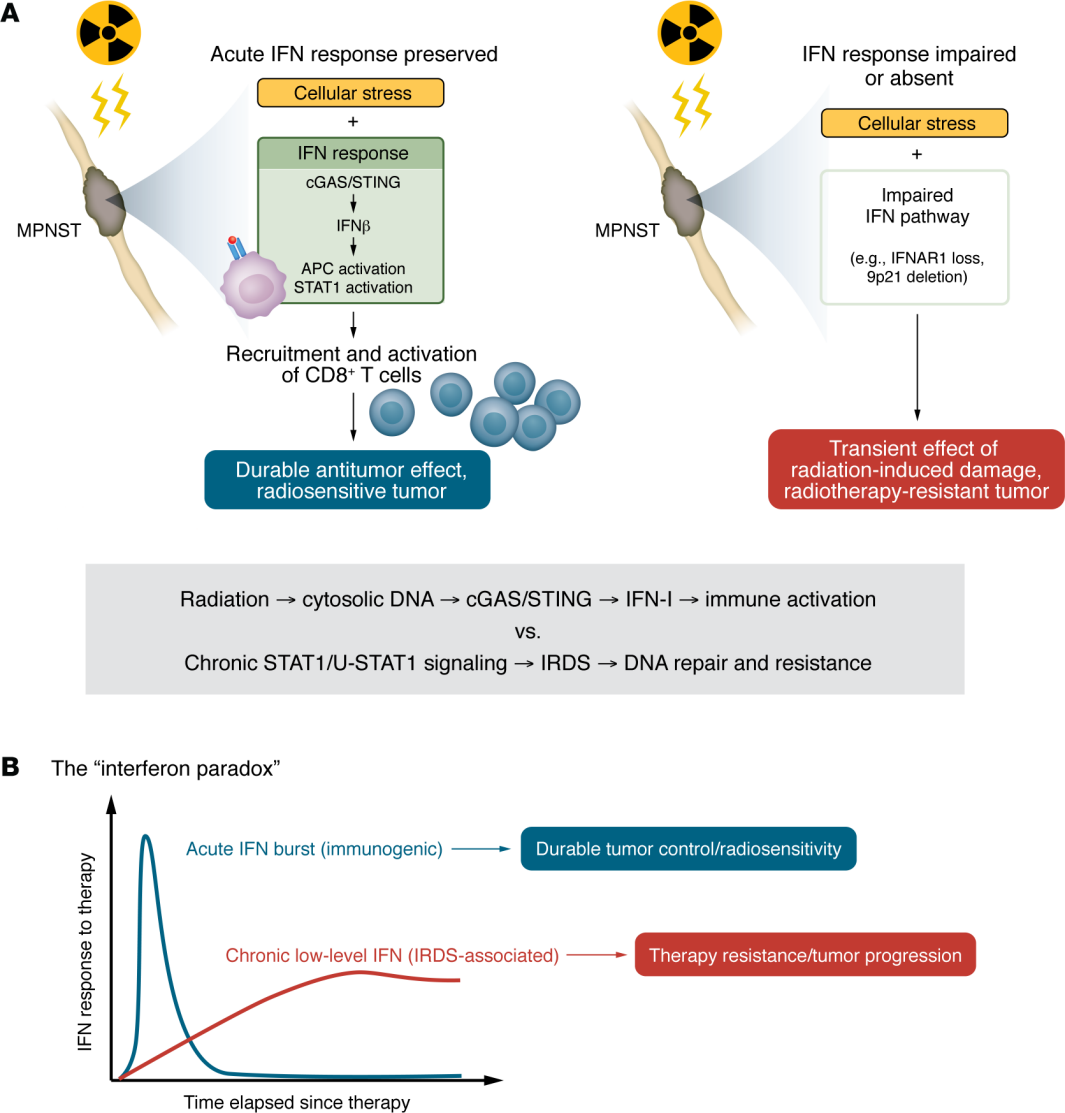
An acute IFN-I response critically determines MPNST sensitivity to radiotherapy. (**A**) Zhu et al. (2) identified the genes encoding STING, IRF3, IFNAR1, and STAT1 as critical determinants of radiosensitivity in human MPNST cell lines. These proteins make up the core signaling axis for the cGAS/STING-driven IFN-I response. Further investigation in murine models of MPNSTs demonstrated that T cell infiltration into tumors, driven by an intact IFN-I response, was necessary for a durable antitumor response to radiation. The findings support a model in which radiation-induced damage induces cytosolic DNA release that triggers cGAS/STING signaling, and the ensuing cGAS/STING-mediated acute IFN-I signaling is central to coordinating immune activation that can drive antitumor effects. Moreover, work from our group and others (3–7) indicates that, by contrast, chronic IFN signaling initiated by STAT1 is associated with an IRDS as well as resistance to radiation and other genotoxic therapies. (**B**) The “interferon paradox” brings together observations that acute exposure to IFN-I signaling has antitumor effects, whereas chronic low-level IFN signaling can enhance cancer cell survival. The paradox provides a framework for resolving conflicting findings related to the role of IFN in therapy resistance.

The most striking findings were observed in immunocompetent mouse models. In mice bearing murine MPNSTs, radiotherapy increased CD8^+^ T cell infiltration into tumors and led to a shift in tumor-associated macrophages toward a more inflammatory, potentially antitumor phenotype. Importantly, when tumors were implanted in T cell–deficient hosts, radiotherapy lost its therapeutic benefit. The radiation still damaged tumor cells; however, it failed to produce a durable response without T cells present to consolidate that initial injury (Figure 1A).

Similarly, when IFNAR1 was deleted specifically in tumor cells in the immunocompetent mice, the radiation response was attenuated even in the presence of an otherwise intact immune system. These findings position IFN signaling as a bidirectional communication system between tumor cells and the immune microenvironment. Radiation does not act in isolation on tumor cells. Rather, it triggers an IFN-mediated dialogue: tumor cells experience DNA damage, produce IFNs, and thereby recruit and activate immune cells that complete the work radiation began. Without this bidirectional signaling — that is, without IFN competence in tumor cells or T cells to receive the signal — radiation failed to produce durable responses, and tumors escape (Figure 1A). These results are consistent with previously published work on the effects of radiation-induced IFN on the immune system.

## Radiotherapy and the IFN paradox

Observations that acute exposure to IFN-I produces growth arrest and apoptosis in cancer cells and, conversely, that chronic low-level exposure can enhance cancer cell survival led to led to the concept of the “interferon paradox” (Figure 1B) (3). This concept provides a framework for understanding the complexities of IFN signaling in cancer. As we will detail below, in the context of radiotherapy, IFN signaling becomes sustained at chronic, low levels, often through tumor cell–intrinsic mechanisms. Tumor cells adapt by upregulating stress response and DNA repair programs; furthermore, the response to chronic IFN signaling may contribute to systemic immunosuppression. In this state, IFN no longer promotes immune-mediated tumor control. Instead, it supports tumor survival by driving resistance mechanisms. The tumor has essentially co-opted an immune danger signal into a survival pathway.

Work from our group by Khodarev and colleagues (4–6) and by Stark and colleagues (3,7) demonstrated that fractionated radiation activates STAT1, which initiates chronic IFN signaling and drives the IFN-related DNA damage resistance signature (IRDS), a transcriptional program that confers resistance to genotoxic therapies including radiation (8). The translational importance of this observation was demonstrated in patients with breast cancer, for whom the IRDS was associated with poorer outcomes and resistance to chemotherapy and radiotherapy. Stark and colleagues refined these observations, noting that unphosphorylated STAT1 (U-STAT1) mediates activation of the IRDS (reviewed in ref. 3). In the context of cellular effects, these findings appear to contrast with the observations of Zhu et al. However, the mechanisms of resistance depend on both initial upstream signals and downstream targets of STAT1, which likely accounts for the heterogeneity of response in human tumors.

Burnette et al. (9) and Deng et al. (10) demonstrated the requirement for IFN signaling in the host for optimal responses to radiation through activation of the cyclic GMP-AMP synthase/stimulator of IFN genes (cGAS/STING) pathway. Radiation produces an acute burst of type I IFN through pathways such as cGAS/STING, which is triggered by cytosolic DNA from damaged tumor cells. In this acute phase, IFN functions as a danger signal. It promotes antigen presentation, activates DCs and other antigen-presenting cells, and supports the recruitment and effector function of CD8^+^ T cells. This is the productive IFN response, one that converts local radiation damage into systemic immune recognition. These findings have been replicated by many investigators, and the findings of Zhu et al. are consistent with these observations.

Zhu and colleagues position MPNSTs within the IFN paradox framework. A therapeutically effective radiation response in these tumors depends on their capacity to mount a timely, immunogenic IFN response rather than maintaining IFN signaling chronically. MPNSTs that cannot generate this acute burst, whether because they lack functional STING/IRF3/STAT1 circuitry or because they have lost IFN gene clusters through chromosomal deletion, fail to recruit immune cells effectively and thereby fail to respond durably to radiation.

The authors extended these mechanistic findings to human MPNST specimens, where they found that low tumor purity, meaning high stromal and immune content, correlates with improved local control following radiotherapy. The authors surmised that chromosome 9p21 loss and the associated loss of the type I IFN gene cluster in that region may contribute to better outcomes in low-purity tumors. In our view, the better outcomes may result from fewer tumor clonogens observed in these tumors, rather than, or in addition to, the loss of chromosome 9p21, although both explanations remain speculative.

## Looking forward: leveraging IFN dynamics in therapeutic design

The central translational implication of this work is that therapeutic strategies should favor acute IFN activation while avoiding chronic IFN signaling. This is easier said than done. We currently lack reliable methods to distinguish these states prospectively in patients, and we do not yet know whether chronic IFN signaling in MPNSTs precedes treatment or emerges as a consequence of therapeutic pressure.

One attractive possibility is to combine radiation with agents that amplify the acute IFN burst; e.g., STING agonists or epigenetic modifiers that enhance IFN pathway transcription (11). The goal of these adjuvant approaches would be to increase the magnitude and immunogenicity of the IFN response without tipping into chronic activation. Conversely, in tumors with evidence of chronic IFN signaling and an IRDS, it may be necessary to transiently suppress IFN pathways during radiation and then reinitiate them afterward to avoid resistance while preserving immune activation.

## Conclusion

In this study, Zhu and colleagues (2) provide a mechanistic foundation for understanding why certain MPNSTs respond to radiotherapy and others do not. Their work highlights how tumor cells and the immune microenvironment cooperate to shape the radiation response, converging on IFN signaling as a central organizing axis. For clinicians managing patients with MPNSTs, this study suggests that future biomarker development should focus not only on tumor cell–intrinsic features but also on immune contexture and IFN pathway integrity. For the broader field of radiation oncology, the findings reinforce an emerging principle: radiation works best when it can engage the immune system, and IFN signaling is often the bridge that makes that engagement possible. Distinguishing productive, acute IFN responses from maladaptive, chronic signaling may guide patient selection and enable more rational combinations of radiotherapy and immunotherapy in MPNSTs and beyond.

## Funding support

This work is the result of NIH funding, in whole or in part, and is subject to the NIH Public Access Policy. Through acceptance of this federal funding, the NIH has been given a right to make the work publicly available in PubMed Central.

- Ludwig Cancer Research Foundation.
- NIH grant 5R01CA262508-05.
- Varian and Regeneron grants (to RRW).
- NIH grants R01CA262508 and U54 CA274291 (to RRW).
- LUNGevity Foundation grant (to SPP).
- Cancer Research Foundation grant (to SPP).
- Falk Medical Research Trust grant (to SPP).
- American Lung Association grant (to SPP).

## Version 1 03/02/2026

Electronic publication
